# MOTIVATION TO LEARN, LITERACY, NUMERACY, AND LATER-LIFE ADULT EDUCATION PARTICIPATION IN THE U.S.

**DOI:** 10.1093/geroni/igz038.2457

**Published:** 2019-11-08

**Authors:** Takashi Yamashita, Phyllis Cummins, Roberto J Millar, Shalini Sahoo, Thomas J Smith

**Affiliations:** 1 University of Maryland, Baltimore County, Baltimore, Maryland, United States; 2 Scripps Gerontology Center, Miami University, Oxford, Ohio, United States; 3 University of Maryland, Baltimore, Baltimore, Maryland, United States; 4 Northern Illinois University, DeKalb, Illinois, United States

## Abstract

The objective of this study is to examine the associations between the motivation to learn, basic skills (i.e., literacy and numeracy), and organized formal and non-formal adult education and training (AET) participation among middle-aged and older adults in the U.S. Rapid technological advancement and globalization requires that adults engage in lifelong learning to actively participate in society. However, little is known about the roles of motivation to learn and basic skills in AET participation among aging adults in the U.S. We obtained restricted data from the 2012/2014 Program for International Assessment of Adult Competencies, and included adults aged 50 years and older (n = 2,580) in the analysis. Structural equation models were used to examine (1) any AET, (2) formal AET and (3) non-formal AET participation as a function of the latent construct of motivation to learn, literacy and numeracy scores (0 – 500), and covariates. Per the confirmatory factor analysis, the motivation to learn latent construct was a valid measure among the older adults. Results from the structural equation models showed that the motivation to learn (b = 0.35, p < 0.05), literacy (b = 0.05, p < 0.05) and numeracy (b = 0.03, p < 0.05) are all positive predictors of non-formal AET participation. However, only motivation to learn (b = 0.47, p < 0.05) is associated with formal AET participation. Findings from this study inform future interventions as well as policy changes to promote specific types of organized AET programs among the aging populations in the U.S.

